# Consumer Acceptability and Community Perceptions of Indigenous Crop-Enriched Stiff Pap in Rural KwaZulu-Natal, South Africa: Implications for Sustainable Food System Transformation

**DOI:** 10.3390/foods15142489

**Published:** 2026-07-14

**Authors:** Sesethu Samuel Ntlanga, Lelethu Mdoda, Denver Naidoo, Laurencia Govender

**Affiliations:** 1 Discipline of Agricultural Economics, School of Agriculture and Science, College of Agriculture, Engineering and Science, University of KwaZulu-Natal, Pietermaritzburg 3209, South Africa; samuelntlanga@gmail.com (S.S.N.); mdodal@ukzn.ac.za (L.M.); 2 African Centre for Food Security (ACFS), School of Agriculture and Science, College of Agriculture, Engineering and Science, University of KwaZulu-Natal, Pietermaritzburg 3201, South Africa; naidook12@ukzn.ac.za; 3 Discipline of Dietetics and Human Nutrition, School of Health Sciences, College of Health Sciences, University of KwaZulu-Natal, Pietermaritzburg 3209, South Africa

**Keywords:** neglected underutilized crops, traditional food, sensory evaluation, consumer acceptability, focus group discussions, indigenous food, food sustainability, rural

## Abstract

Indigenous crops are well adapted to marginal conditions and rich in nutrients, making them promising contributors to food and nutrition security in rural South African communities. This study evaluated the consumer acceptability and community perceptions of stiff pap composite dishes incorporating pumpkin leaves, *Cucurbita* pumpkin, and cream-fleshed sweet potato (CFSP) among 60 rural participants. Employing a cross-sectional design, sensory testing using a nine-point hedonic scale and focus group discussions (FGDs) were conducted across the uMkhanyakude and King Cetshwayo District Municipalities in KwaZulu-Natal, South Africa. All three composite dishes were well-liked overall. The CFSP-based dish achieved the highest overall acceptability (7.68) and was the most preferred (40%; n = 24), while the pumpkin-based dish was the least preferred (38%; n = 23). Significant differences in taste and color were observed across dishes (*p* < 0.05). Focus groups highlighted that familiarity, flavor balance, preparation methods, and cultural norms shaped willingness to adopt these dishes, with novel combinations eliciting both curiosity and hesitation. Cultural norms, family preferences, and the traditional significance of stiff pap shape acceptance pathways. The findings suggest that integrating indigenous crops into culturally familiar staples can promote dietary diversification and support smallholder farming systems, with culturally sensitive culinary guidance serving as the key enabler for broader adoption. These findings imply that embedding indigenous crops within culturally central staples offers a practical, consumer-driven entry point for sustainable food system transformation, simultaneously advancing dietary diversity, smallholder livelihoods, and the resilience of rural food systems.

## 1. Introduction

The prevailing global food system is characterized by a fundamental failure, namely its inability to deliver food security and nutritional adequacy in a sustainable and equitable manner. This failure is evident in sub-Saharan Africa (SSA), a region where agricultural systems are simultaneously confronting converging pressures from climate change, environmental degradation, and rapid population growth [1]. This narrow agrobiodiversity has created a fragile food supply vulnerable to climatic shocks and market volatility, and has led to hidden hunger, a form of micronutrient malnutrition that affects over two billion people globally [1,2].

Neglected and underutilized crops (NUCs), also referred to as orphan or indigenous crops, offer a potential pathway to address these interconnected challenges. These species are typically nutrient-dense, drought-tolerant, and adapted to marginal soils, requiring fewer external inputs than conventional staples such as maize and wheat. Indigenous crops have sustained rural communities for generations and remain integral to traditional food systems and cultural practices [3,4,5]. Despite this, NUCs remain marginalized within dominant food systems. Cultural perceptions that stigmatize them as ‘poor people’s food’ suppress both demand and farmer motivation [3,6]. As documented across SSA, the very chemical compounds responsible for NUCs’ significant health benefits simultaneously create substantial sensory challenges that limit consumer acceptance, presenting a fundamental dilemma for product development and commercialization efforts [4,7].

Smallholder farming remains integral to rural livelihoods in South Africa, providing both food and income to millions of households [8,9,10,11]. In KwaZulu-Natal (KZN), uMkhanyakude and King Cetshwayo District Municipalities are characterized by predominantly rural populations that rely heavily on subsistence agriculture, with significant levels of food insecurity and malnutrition [12,13]. These districts are recognized for their agroecological diversity, supporting traditional crops including indigenous vegetables (*imifino*), traditional grains (sorghum and finger millet), drought-tolerant legumes, and indigenous fruits. Among smallholder farmers surveyed in these districts, approximately 40% have adopted NUC cultivation, driven primarily by traditional knowledge and cultural affiliation rather than formal agricultural extension [14].

In rural South Africa, stiff pap (locally known as *phutu* or *sadza*), traditionally made from refined maize meal, is the predominant staple food for most households due to its low cost, satiety, and deep cultural embeddedness [15]. However, maize-based stiff pap is nutritionally poor, providing primarily energy-dense starch with limited protein, dietary fiber, and essential micronutrients. Enriching stiff pap with indigenous crops presents a promising, culturally grounded strategy for improving rural diets. By incorporating indigenous crops such as cream-fleshed sweet potatoes, traditional *Cucurbita* pumpkins, and pumpkin leaves, it becomes possible to enhance the nutritional density of a widely consumed staple without displacing familiar eating practices [15,16].

The three indigenous crops examined here differ markedly in their nutritional contributions. Pumpkin leaves are a particularly nutrient-dense leafy vegetable: an average portion makes a substantial contribution to the recommended dietary allowance (RDA) for several micronutrients, supplying close to half of the adult RDA for iron (49%) and vitamin A (44%), roughly a fifth to a quarter for calcium (22%), magnesium (21%), and copper (22%), and around 30% for manganese, while remaining low in energy [17,18]. Their high iron content makes pumpkin leaves a valuable plant source of this mineral relative to other African leafy vegetables, and they are also rich in folate and dietary fiber [17,18]. *Cucurbita* pumpkin flesh is a good source of provitamin A carotenoids, tocopherols, dietary fiber, and minerals such as potassium, phosphorus, and magnesium, and is low in energy [19]. It also carries recognized health-promoting properties: pumpkin has been widely used for its antioxidant, anti-inflammatory, and antidiabetic activity, and is reported to help regulate blood glucose, in part through reduced α-glucosidase activity and enhanced insulin response [19]. Cream-fleshed sweet potato (CFSP), in turn, contributes readily digestible energy together with a soft texture and natural sweetness that enhance palatability, along with modest amounts of protein, dietary fiber, and minerals such as potassium, iron, and zinc [20]. Unlike orange-fleshed varieties, however, cream-fleshed cultivars contain only negligible provitamin A (β-carotene) [20], so within a composite dish their main contribution lies in energy, texture, and consumer acceptability rather than vitamin A density. In rural South African households, these crops are most commonly prepared by boiling: pumpkin leaves are cooked as a leafy relish (*imifino*) to accompany a starchy staple, while pumpkin and sweet potato are boiled, mashed, or incorporated into bean-based side dishes. Beyond these traditional preparations, the crops can also be processed into flours, purees, and dried products for incorporation into composite staples such as the enriched stiff pap evaluated in this study, broadening the range of food products in which they can be used.

However, the successful adoption of such composite foods depends critically on consumer acceptability. This acceptability is a multidimensional construct that extends well beyond sensory evaluation alone: it integrates the hedonic sensory response to a food’s taste, texture, aroma, color, and appearance; the cultural and social acceptability of the modified dish within established household foodways; and the behavioral willingness of consumers to prepare and repeatedly serve the food to their families. Sensory liking is therefore a necessary but not sufficient condition for acceptance, and in the present study, it is deliberately complemented by community perceptions and stated willingness to adopt, captured through focus group discussions. Modifying a traditional dish like stiff pap introduces potential sensory and cultural challenges. Indigenous crops can alter the taste, texture, color, and aroma of the final product, which may be met with resistance, especially among younger consumers who may associate indigenous ingredients with old-fashioned or less desirable rural diets [7]. Sensory evaluation studies reveal that unfamiliar taste profiles, including earthy, bitter, or astringent notes common in many NUCs, and deeply ingrained texture preferences for refined staples, create substantial psychological and practical barriers to adoption [4]. Understanding these sensory perceptions and cultural barriers is therefore as important as confirming nutritional benefits.

Despite these agronomic and nutritional advantages, evidence on how rural consumers actually perceive, sensorially evaluate, and choose to adopt indigenous-crop-rich staples remains limited in the South African context, and it is precisely this shortage of consumer-level evidence that provides the rationale for the present work.

As urbanization and dietary transitions reshape food choices, generating evidence on consumer preferences is increasingly important for mainstreaming indigenous foods. For smallholder farmers, developing acceptable composite pap recipes also offers opportunities for value addition. Processing indigenous crops into enriched stiff pap can create new income streams and support sustainable farming systems. Enhancing consumer markets for indigenous crop-based foods incentivizes diversification and resilience against climate variability [16,21,22].

Hence, this study examines consumer acceptability and community perceptions of stiff pap composite dishes prepared with indigenous crops cultivated by smallholder farmers. By evaluating sensory attributes and consumer attitudes through both quantitative sensory evaluation and qualitative focus group discussions, the research aims to inform product development, nutrition promotion, and value chain enhancement within a sustainable food systems framework. Ultimately, it contributes to revitalizing indigenous crops, strengthening smallholder livelihoods, and promoting culturally relevant, nutrient-dense diets to improve food and nutrition security in rural South African communities.

## 2. Materials and Methods

### 2.1. Schematic Overview of the Experimental Program

**Figure 1:**
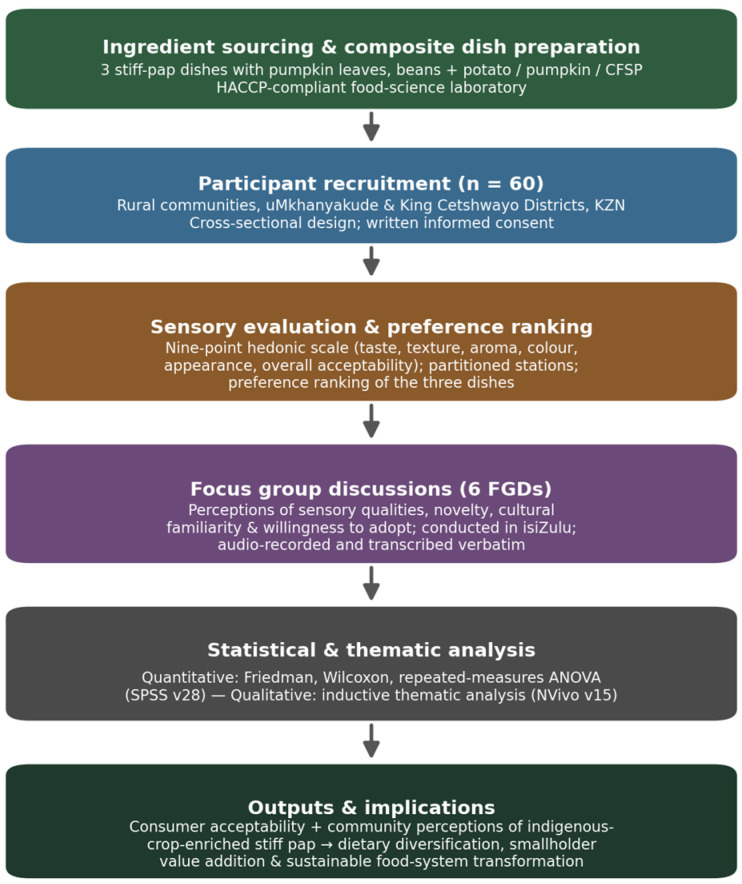
Schematic overview of the experimental programme.

Figure 1 presents a schematic overview of the research methodology followed in this study, encompassing ingredient sourcing and composite-dish preparation, participant recruitment, sensory evaluation and preference ranking, focus group discussions, and the combined statistical and thematic analysis. The diagram links each methodological step to the work’s central objective: assessing the consumer acceptability and community perceptions of indigenous-crop-enriched stiff pap among rural consumers. This overview is directly relevant to sustainable food system transformation because it makes explicit how smallholder-produced indigenous crops, a culturally central staple, and consumer-level evidence are connected within a single pathway toward dietary diversification, strengthened local value chains, and more resilient rural food systems. The procedure at each stage followed standard, previously published protocols, Hazard Analysis and Critical Control Point principles for dish preparation [23], the nine-point hedonic scale for sensory evaluation [24], and inductive thematic analysis for the focus group discussions [25], to ensure the authenticity, reproducibility, and validity of the methodology.

### 2.2. Materials

**Figure 2:**
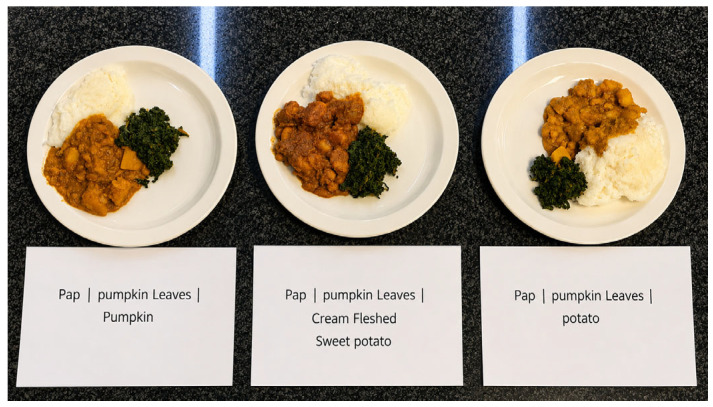
Composite dishes.

Three composite dishes were prepared for this study. Each dish comprised stiff pap served alongside pumpkin leaves and a variation of bean curry. Composite Dish 1 was a stiff pap accompanied by pumpkin leaves, beans, and potatoes (traditionally served with a combination of beans and potatoes). Composite Dish 2 was stiff pap served with pumpkin leaves, beans, and pumpkin (*Cucurbita* spp.). Composite Dish 3 was stiff pap served with pumpkin leaves, beans, and cream-fleshed sweet potato (CFSP) (Figure 2).

**Figure 3:**
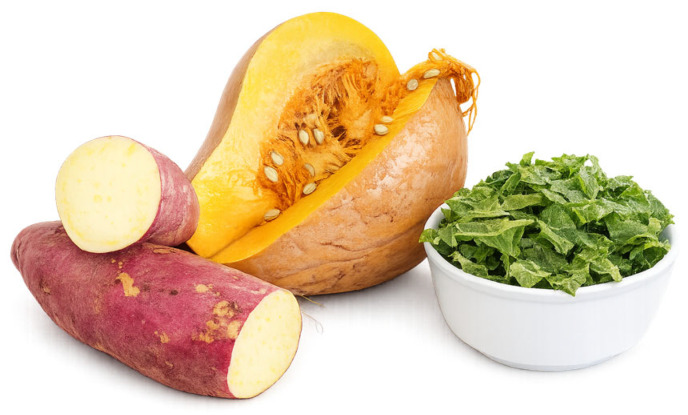
Raw ingredients (Sweet potato, pumpkin and pumpkin leaves).

Commercially available components, including Nyala maize meal, red speckled dry beans, onions, potatoes, Robertson’s Rajah Mild spice, Cerebos salt, Knorrox beef stock cubes, Robertson’s Rajah curry powder, cooking oil, and cayenne pepper, were acquired from a local supermarket in Pietermaritzburg, KZN, South Africa. Nyala maize meal was selected for its status as the most widely utilized brand in the province. Pumpkin leaves and pumpkin (*Cucurbita* spp.) (Figure 3) were donated by smallholder farmers from Swayimane village, while the cream-fleshed sweet potatoes (Figure 3) were procured from local fruit and vegetable supermarket at Scottsville Mall in Pietermaritzburg, KZN, South Africa.

### 2.3. Study Design

A cross-sectional study design was utilized to assess the sensory attributes and participants’ perceptions of dishes prepared from indigenous crops, specifically cream-fleshed sweet potatoes, *Cucurbita* pumpkin, and pumpkin leaves, which are commonly cultivated in resource-limited regions of KZN. The research was conducted at the University of KZN within the Msunduzi Local Municipality. Standardized dishes were prepared, and participants from rural communities in uMkhanyakude District Municipality (UDM) and King Cetshwayo District Municipality (KCDM) in KZN were invited to participate in a one-time sensory evaluation, followed by focus group discussions. Data were collected at a single time point to capture participants’ acceptability, perceived quality, and related outcomes, thereby providing insights into both individual sensory responses and broader community perceptions of dishes based on indigenous crops. These two districts were purposively selected due to their topographical suitability for NUC production, documented food security challenges, predominantly rural populations relying on subsistence agriculture, and the documented presence of traditional crop cultivation [12,13].

### 2.4. Ethical Considerations

The study was conducted in accordance with the Declaration of Helsinki, and approved by the University of KwaZulu-Natal Humanities and social sciences Research Ethics Committee (HSSREC/00007019/2024). Written informed consent was obtained from all participants prior to their involvement in the study. Participation was entirely voluntary. In addition, prior to participation in the sensory evaluation, preference ranking, and focus group discussions, written informed consent was obtained from all participants, with consent forms made available in both English and isiZulu.

### 2.5. Preparation of Composite Dishes

All composite dishes were prepared freshly on the morning of the sensory evaluation data collection, which took place in the Food Processing Laboratory of the Human Nutrition and Dietetics Department at UKZN, Pietermaritzburg. All ingredients were processed and prepared in a certified Food Science laboratory in accordance with Hazard Analysis and Critical Control Point (HACCP) principles [23] to ensure food safety, minimize contamination risks, and preserve the integrity of the composite dishes. The food items were prepared over two trial sessions, one week prior to the pilot study, to ensure that the recipes were both accurate and culturally acceptable. The dishes were tasted by Black African men and women employed at UKZN, who shared a similar sociodemographic background with the study participants, to assess cultural acceptability prior to the main data collection.

### 2.6. Sensory Evaluation

A pilot study was conducted at the University of KZN to evaluate the feasibility of the study procedures and the preliminary consumer acceptability of the newly developed recipes. Three composite meal combinations were prepared and assessed. Each of the three dishes was assigned a three-digit code using a random-number table [26], with the code known solely to the researcher and the research assistants. Ten participants from KwaNongoma village in northern KZN were recruited for this pilot. Prior to participation, informed consent was obtained, and participants completed the sensory evaluation, preference ranking test, and focus group discussions. These pilot participants were subsequently excluded from the main study.

**Figure 4:**
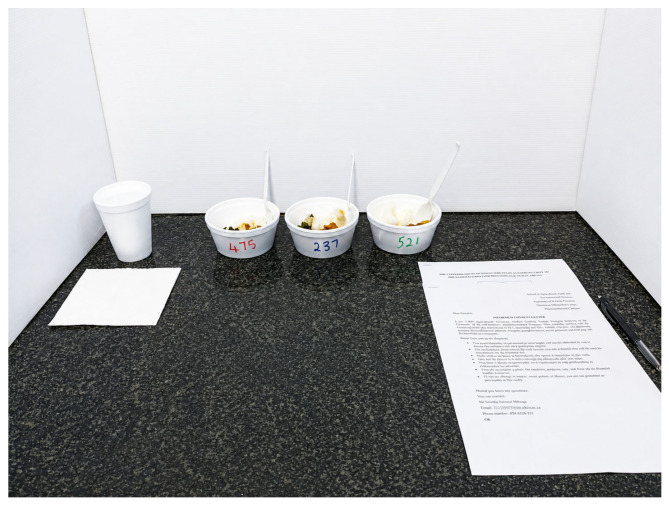
Sensory setup: three coded samples, scoresheet, pen, serviette, and palate-cleansing water.

For the main sensory evaluation, 60 untrained participants (UDM and KCDM) were randomly selected from those available at the time of the study to assess the acceptability of the three composite dishes. To mitigate potential bias and prevent mutual influence among panellists, participants were seated at physically separated stations, partitioned by boards, and instructed to refrain from communication during the sensory evaluation sessions. Figure 4 illustrates the sensory evaluation set-up.

At the commencement of each session, the researcher guided the panellists through the evaluation forms to eliminate errors and inaccuracies, using IsiZulu for clarity. Individual tasting stations were established for each participant, featuring coded food samples and a glass of water to facilitate palate cleansing between samples [26].

Each participant received the heated samples and was instructed to assess the acceptability and rank the dishes using a nine-point hedonic scale (1 = extremely dislike to 9 = like extremely) [24]. Participants then indicated their preference order among the three dishes: 1 = most preferred, 2 = moderately preferred, and 3 = least preferred. It should be noted that the sensory panel was strongly concentrated among young adults, with 80% of participants aged 18 to 25 years. While this reflects the random selection of available community members who consented to participate, it constitutes a methodological limitation, as the panel does not fully represent the age structure of the entire rural community, and the acceptability scores may be more reflective of younger consumers’ preferences than those of older consumers.

### 2.7. Focus Group Discussions Method

Following the sensory evaluation, participants participated in six focus group discussions (FGDs). Each FGD had ten participants and was facilitated by a trained individual with experience in conducting focus groups [27]. The questions were developed in advance and validated through the pilot study. The FGDs explored participants’ perceptions of taste, texture, color, and aroma, their willingness to prepare these dishes for their families, and the cultural and social factors shaping adoption decisions. Each session lasted approximately 30 min and was conducted entirely in isiZulu, the participants’ home language, by a facilitator fluent in isiZulu. Discussions were held in a quiet, familiar communal setting at the data-collection venue, and participants were encouraged to speak freely in their own language to ensure that rural participants felt comfortable and could express their views openly. With participants’ consent, all sessions were audio-recorded and subsequently transcribed verbatim and translated into English for analysis.

### 2.8. Statistical Analysis

Quantitative data were systematically coded, recorded in Microsoft Excel, and meticulously cleaned prior to export to the Statistical Package for the Social Sciences (SPSS) program (Statistical Package for Social Science version 28, SPSS Inc., Chicago, IL, USA) for subsequent statistical analysis. The Wilcoxon rank-sum test and Friedman test were used to compare sensory attributes across dishes, with the Bonferroni correction applied for pairwise comparisons. Preference rankings were analyzed using repeated measures ANOVA. Qualitative data derived from the FGDs, facilitated by a semi-structured discussion guide, were audio-recorded, transcribed verbatim, and analyzed utilizing NVivo qualitative data analysis software (NVivo version 15, Lumivero, Denver, CO, USA) through inductive thematic analysis.

## 3. Results

### 3.1. Sample Characteristics

Table 1 summarizes the sample characteristics of the 60 study participants. Females constituted the majority of the sample (n = 39, 65.0%), while males constituted the minority (n = 21, 35.0%). The 18-to-25-year age group was the most represented, accounting for 80.0% (n = 48) of participants. Smaller proportions were observed in the 26-to-35-year (n = 4, 6.7%), 36-to-45-year (n = 1, 1.7%), 46-to-55-year (n = 4, 6.7%), and 56-to-65-year (n = 3, 5.0%) age categories.

### 3.2. Consumer Acceptability

The mean sensory evaluation scores for the three composite dishes across the six attributes measured on a nine-point hedonic scale are presented in Table 2. The *t*-test analysis indicated that sensory evaluation scores for each attribute were above the neutral score, suggesting that, on average, all composite dishes were well-liked. The Wilcoxon rank test revealed that, while the stiff pap with pumpkin leaves and beans, accompanied by pumpkin, received a significantly lower aroma rating (6.75), the overall acceptability was rated high (7.17). The ratings for texture (7.12), appearance (7.02), and color (6.92) were significantly lower than the overall acceptability for the composite dish made with stiff pap, pumpkin leaves, beans, and potato. Furthermore, aroma (6.97), appearance (6.95), and color (6.90) of the composite dish comprising stiff pap, pumpkin leaves, and beans with CFSP were rated significantly lower than the overall acceptability (7.68). Results from the Friedman test indicated that taste and color differed significantly across the three composite dishes (*p* < 0.05). The Bonferroni correction showed that the taste of the stiff pap, pumpkin leaves, and beans with the CFSP dish had a higher mean rank than those with the pumpkin dish.

Analysis from a repeated measures ANOVA confirmed that the mean preference rankings did not differ significantly across the three dishes (*p* = 0.284). However, it is numerically evident that the combination of stiff pap, pumpkin leaves, and beans with CFSP was the most preferred (n = 24; 40%), while the combination of stiff pap, pumpkin leaves, and beans with pumpkin was the least preferred (n = 23; 38%).

### 3.3. Focus Group Discussions

Table 3 presents the results of the FGDs, summarizing participants’ perceptions towards the three composite dishes across key discussion questions. Thematic analysis identified recurring concepts relating to sensory qualities, novelty, cultural familiarity, and adoption readiness.

## 4. Discussion

The socio-economic landscape of South Africa presents a complex web of structural inequalities and systemic challenges that have profoundly exacerbated household food accessibility, particularly in rural and peri-urban communities. The Gini coefficient in South Africa is estimated at 0.68, indicating one of the highest levels of income inequality globally, which directly undermines the ability of low-income households to afford nutritious diets [28]. Evidence indicates that under such circumstances, households increasingly depend on staple-based diets and locally produced, affordable, and culturally familiar foods [8,11]. While this adaptive strategy may facilitate short-term food availability, it frequently results in nutritionally monotonous diets, thereby heightening vulnerability to micronutrient deficiencies and inadequate protein quality.

The pronounced concentration of participants in the 18-to-25-year age group (80.0%; n = 48) (Refer to Table 1) represents a limitation of the present sample. Because young adults dominated the panel, the acceptability ratings and preference rankings reported in Section 3.2 should be interpreted as reflecting predominantly the responses of younger consumers rather than the full age spectrum of the rural community. Younger rural residents are more exposed to urban dietary transitions and may differ systematically from older community members in their familiarity with, and openness to, indigenous-crop combinations. Consequently, the findings may not fully capture the perceptions of older adults, for whom traditional indigenous dishes are often more deeply embedded in everyday eating practices.

This demographic profile is broadly consistent with the wider study population, where 68.5% of NUC adopters are female, and adopters are older on average (mean age 52.8 years) than the predominantly younger sensory panel, reflecting the different recruitment purposes of the two samples [14]. This age gap is not merely an artifact of sampling; it has substantive implications for how acceptability should be interpreted. Younger rural residents are more exposed to the urban dietary transition, characterized by greater consumption of refined, commercially marketed, and convenience foods, and are more likely to associate indigenous-crop dishes with old-fashioned or low-status rural diets [3,7]. Their food preferences are therefore shaped by aspirational, market-mediated tastes and a comparatively weaker habitual exposure to traditional preparations [7]. Older residents, by contrast, typically retain stronger experiential familiarity with indigenous crops, anchoring their acceptance in cultural memory, established preparation routines, and intergenerational food practices rather than novelty [3,29]. As a result, the favorable ratings obtained from this predominantly young panel may understate the acceptability that would be observed among older community members for whom these dishes are already culturally embedded, while at the same time providing a more conservative and arguably more demanding test of whether indigenous-crop-enriched stiff pap can appeal to the generation most responsible for shaping future household diets. This structural difference should therefore be weighed when extrapolating the present findings to the rural community as a whole.

Consumer acceptability results provide insights into the feasibility of translating nutritional potential into practical dietary applications. All three composite dishes received favorable ratings overall, indicating that nutrient-enhanced versions of stiff pap are acceptable within the study population. The stiff pap with pumpkin leaves, beans, and CFSP emerged as the most preferred option (Refer to Table 2). This preference appears to be influenced by sensory attributes, particularly the natural sweetness and softer mouthfeel associated with CFSP. Sensory satisfaction is a crucial determinant of food choice, especially among younger adults, and the present findings reinforce the need to align nutritional interventions with positive eating experiences. This finding is consistent with evidence across SSA showing that consumer acceptance of NUC-based products is primarily driven by taste and texture, with unfamiliar flavor profiles creating a significant novelty barrier that limits market penetration and commercial viability [4].

Considering the attribute-level scores in Table 2 in more detail, the CFSP-based composite dish recorded the highest mean ratings for taste (7.65) and overall acceptability (7.68), as well as the highest texture score (7.38), which is consistent with the natural sweetness and softer mouthfeel of cream-fleshed sweet potato. The potato-based composite dish achieved comparably high overall acceptability (7.48) and the highest aroma rating (7.37), whereas the pumpkin-based composite dish recorded the lowest taste (6.90), aroma (6.75), and overall acceptability (7.17) scores but, notably, the highest color rating (7.33). Across all three dishes, individual attribute means clustered between approximately 6.75 and 7.68 on the nine-point scale, indicating that every attribute was rated above the neutral midpoint and that differences between dishes, although statistically significant for taste and color (*p* < 0.001, Friedman test), were modest in absolute terms. This pattern indicates that the comparative advantage of the CFSP dish was driven primarily by taste and texture rather than by appearance-related attributes, for which the pumpkin dish performed best.

The pumpkin-and-beans combination, despite its nutritional robustness documented in the companion article, was perceived by many participants as unfamiliar. Focus group discussions revealed that limited prior exposure contributed to initial hesitance, particularly concerning flavor compatibility and texture. Importantly, this unfamiliarity did not result in outright rejection; several participants expressed curiosity and openness to experimenting with the combination, suggesting that acceptability may improve with repeated exposure and refined preparation methods. This finding is consistent with observations that the declining consumption of indigenous foods is often attributed to evolving food habits rather than dislike [7]. Perceptions and attitudes of smallholder farmers toward NUCs are significantly influenced by traditional knowledge, cultural affiliation, and awareness, with adopters showing more positive orientations toward NUCs (mean perception index = 0.76) compared to non-adopters [14]. The novelty associated with pumpkin-and-beans combinations should therefore be regarded as an opportunity for gradual dietary diversification rather than a limitation.

Texture-related concerns, particularly regarding pumpkin leaves, were among the most frequently cited barriers to acceptance. These findings emphasize the significant role of food preparation practices in shaping both sensory quality and consumer response. Sensory evaluation studies have documented that extended cooking times and characteristically dense, firm textures in NUC-based products substantially reduce their appeal to modern urban consumers [30]. Addressing these challenges through household-level culinary training and demonstration-based nutrition education may thus enhance both acceptance and nutrient retention. The strategic deployment of culinary demonstrations can enhance brand credibility and accelerate consumer adoption by disseminating knowledge and exerting normative influence [3]. Cultural perceptions and social status associations emerge as formidable barriers to NUC adoption. Multiple studies document the stigmatization of NUCs as ‘poor people’s food’, creating social resistance that frequently outweighs nutritional advantages [3,7,22]. This perception is reinforced by strong associations with poverty and rural life, leading to status-driven rejection, especially among younger consumers. Focus group findings in this study corroborate this dynamic: the “my family is reluctant to change” theme reveals that household-level cultural norms constitute a substantive barrier (Refer to Table 3) that cannot be addressed through nutritional information alone. Systematic rebranding initiatives must consciously dissociate these crops from their historical associations with poverty while stressing their distinctive nutritional and cultural attributes [3].

The bitter and salty aftertastes reported for the cooked pumpkin leaves (Table 3) further illustrate how the very phytochemicals that confer health benefits can simultaneously generate sensory resistance. The polyphenols, tannins, and related bioactive compounds that contribute to the antioxidant and nutritional value of indigenous leafy vegetables also produce the earthy, bitter, and astringent notes that unaccustomed consumers often find unappealing [31], and similar bitterness-driven acceptance barriers have been documented for other indigenous staples such as finger millet, where sensory masking or modified preparation is frequently required to broaden appeal [32]. From a practical standpoint, these aftertastes can be mitigated through established household techniques such as pre-boiling and discarding the first cooking water, brief blanching, careful seasoning, and combining the leaves with naturally sweet components such as cream-fleshed sweet potato, which may partly explain the comparatively higher ratings achieved by the CFSP-based dish. Importantly, the bitterness reported here did not translate into outright rejection, and a substantial proportion of participants still expressed willingness to prepare the dishes, echoing evidence that the declining consumption of African leafy vegetables in KZN is driven more by shifting food habits and the erosion of traditional preparation knowledge, particularly among younger generations, than by intrinsic dislike [29]. This reinforces the case for embedding culinary guidance that revives optimal preparation practices alongside any promotion of indigenous-crop-enriched stiff pap.

The willingness to adopt sweet-potato-and-beans for family cooking, expressed by the majority of focus group participants, signals that sensory acceptability is a plausible pathway to household dietary change. From an agricultural and food security perspective, this acceptability creates demand that could incentivize smallholder farmers to expand NUC cultivation. The capacity to cultivate pumpkin leaves, pumpkin flesh, and associated crops locally diminishes reliance on volatile food markets and bolsters household resilience during periods of economic instability. Enhancing consumer markets for indigenous crop-based foods through value addition and product development can create pathways for improved livelihoods among smallholder farmers while simultaneously mitigating nutritional insecurity [21,22]. An integrated marketing approach that emphasizes both the cultural authenticity and the nutritional value of these foods, incorporating culinary influencers and demonstration cookery events, could reposition indigenous crops as desirable foods rather than poverty foods.

Beyond acceptability, the value of these composite dishes lies in their nutritional complementarity. Maize-based stiff pap is energy-dense but deficient in lysine and other essential amino acids, and the addition of pumpkin leaves and beans, both protein- and mineral-rich, improves the amino acid balance and micronutrient density of the meal without displacing the familiar staple. This combination of a cereal staple with legumes and a nutrient-dense leafy vegetable is a classic food-based strategy for raising protein quality and addressing micronutrient deficiencies in resource-constrained settings, where access to diverse, animal-source protein is limited [33]. From a dietary-diversity perspective, encouraging the routine incorporation of indigenous crops into a single, widely consumed dish offers a pragmatic entry point for broadening nutrient intake at the household level, since it works within existing meal structures rather than requiring wholesale changes to eating patterns. Linking such acceptable, nutrient-enhanced recipes to nutrition-sensitive agricultural programs can therefore align consumer demand, smallholder production, and improved diet quality within the same intervention, provided that promotion is culturally grounded and accompanied by practical preparation guidance [34]. These nutritional and behavioral considerations strengthen the rationale for positioning indigenous-crop-enriched stiff pap as a realistic component of food-based approaches to nutrition security in rural KZN.

Several limitations should be acknowledged when interpreting these findings. First, the study was based on 60 participants drawn from two district municipalities, which, while adequate for an exploratory sensory and perception study, limits the statistical power and the generalizability of the results to the broader rural population of KZN and South Africa. Second, the panel was strongly skewed towards young adults (80% aged 18 to 25 years), so the acceptability ratings may not fully represent the preferences of older community members, among whom indigenous dishes are often more culturally embedded. Third, the cross-sectional design captures acceptability at a single point in time and therefore cannot establish whether initial acceptance translates into sustained adoption or measurable changes in dietary behavior over time. Accordingly, the broader implications drawn here for dietary diversification, food and nutrition security, and the strengthening of smallholder farming systems should be regarded as plausible, evidence-informed pathways rather than directly demonstrated outcomes of this study. Longitudinal, larger, and more demographically representative studies, ideally combining repeated sensory exposure with measures of actual household consumption and procurement, are needed to substantiate these broader claims.

## 5. Conclusions

This study demonstrates that integrating indigenous crops into the culturally central staple, stiff pap, is achievable without compromising consumer acceptability. All three composite dishes received favorable overall ratings, with the stiff pap with pumpkin leaves, beans, and cream-fleshed sweet potato emerging as the most preferred. Although the pumpkin-and-beans combination was less familiar to participants, it demonstrated potential for broader adoption with improved preparation methods and repeated exposure. Focus group discussions revealed that familiarity, flavor balance, and cultural norms around the correct appearance and texture of stiff pap are critical mediators of acceptability. Social dynamics, particularly family resistance to dietary change, present a structural barrier that cannot be overcome through nutritional information alone. These findings highlight the importance of promoting nutrient-dense, culturally familiar composite foods as viable dietary strategies in contexts where households face limited access to diverse food options.

Beyond individual acceptability, the foremost implication of this work is for sustainable food system transformation: embedding smallholder-grown indigenous crops within a culturally central staple demonstrates a scalable, consumer-driven pathway for simultaneously diversifying rural diets, adding value to indigenous-crop value chains, and strengthening the resilience of local food systems. Taken together, indigenous crop-enriched stiff pap can be understood as a culturally embedded vehicle for improving the nutritional quality of a familiar staple without displacing established eating practices. Fortifying a low-cost, energy-dense food with nutrient-rich indigenous crops offers a practical, food-based response to hidden hunger that simultaneously creates demand for crops grown by smallholder farmers. Its future development is likely to advance along three converging trajectories: first, recipe standardization and culinary guidance that optimize sensory quality and nutrient retention while respecting cultural expectations of stiff pap; second, value addition and product development, including indigenous-crop flours, purees, and ready-to-cook composite mixes that link smallholder production to local markets; and third, integration into nutrition-sensitive agriculture and school or community feeding programs that reposition indigenous crops as desirable, health-promoting foods rather than poverty foods. Realizing these trends will depend on culturally grounded promotion, supportive value chains, and continued evidence on consumer acceptability. Consequently, it is recommended that nutrition programs and agricultural extension initiatives facilitate the production and household-level utilization of pumpkin and pumpkin leaves, alongside culinary education that emphasizes enhanced preparation techniques and the gradual introduction of novel food combinations, to bolster food and nutrition security while supporting sustainable smallholder farming systems. Future research should focus on longitudinal acceptability studies to assess whether repeated exposure increases acceptance of the pumpkin-and-beans combination, and on developing targeted culinary training programs that address the specific preparation barriers identified in this study.

## Figures and Tables

**Table 1:** Sample characteristics of the 60 sensory evaluation participants.

Category	n	%
Gender		
Male	21	35.0
Female	39	65.0
Age group (years)		
18 to 25	48	80.0
26 to 35	4	6.7
36 to 45	1	1.7
46 to 55	4	6.7
56 to 65	3	5.0

n = 60 participants.

**Table 2:** Sensory evaluation comparison of the three composite dishes.

Sample	Taste	Texture	Aroma	Color	Appearance	OA
Stiff pap with pumpkin leaves, beans and potatoes	7.45 ± 1.56	7.12 ± 1.67	7.37 ± 1.28	6.92 ± 1.70	7.02 ± 1.55	7.48 ± 1.35
Stiff pap with pumpkin leaves, beans and CFSP	7.65 ± 1.53	7.38 ± 1.48	6.97 ± 1.46	6.90 ± 1.67	6.95 ± 1.55	7.68 ± 1.48
Stiff pap with pumpkin leaves, beans and pumpkin	6.90 ± 1.72	6.95 ± 1.55	6.75 ± 1.57	7.33 ± 1.50	6.97 ± 1.31	7.17 ± 1.39
*p*-value (Friedman)	***p* < 0.001**	***p* < 0.001**	***p* < 0.001**	***p* < 0.001**	***p* < 0.001**	***p* < 0.001**

OA: Overall acceptability; CFSP: cream-fleshed sweet potato. Values are mean ± SD. Scores are based on a nine-point hedonic scale (1 = extremely dislike to 9 = like extremely). Bold *p*-values indicate significance at *p* < 0.05 according to the Friedman test.

**Table 3:** Participants’ perceptions towards the three composite dishes.

Question	Concept	Theme	Codes	Representative Quote	Interpretation
What did you think about the cooked pumpkin leaves?	Sensory qualities determine liking	Mixed perceptions of taste	Salty; Good taste; Delicious; Bitter aftertaste	“Very nice but a bit salty.” (FG2, P1); “Had a bitter after taste.” (FG3, P1)	Taste varied greatly among participants. Saltiness and bitterness were common complaints.
	Texture as a key acceptability driver	Texture influenced liking	Rough texture; Soft; Undercooked	“Texture was a little rough.” (FG3, P2); “Not well cooked.” (FG4, P2)	Rough or undercooked textures caused negative reactions. Well-cooked leaves were highly valued.
	Visual cues influence acceptance	Visual appeal contributed positively	Good color; Appealing appearance	“I liked the color.” (FG4, P10)	Appearance shaped perceptions of quality and positively influenced liking.
What did you think about the addition of sweet potatoes to the beans?	Sweetness enhances acceptance	Widely liked and often the most preferred	Delicious; Favorite; Good aroma; Well-seasoned	“This is my favorite dish.” (FG1, P1); “Most preferred dish.” (FG6, P2)	The sweet potato combination was typically the favorite across groups due to its flavor, sweetness, and aroma.
	Novel combinations can surprise positively	Surprise at the combination	Unexpected; New experience; Balanced sweetness	“I was not aware that sweet potato can be mixed with beans.” (FG1, P2)	Novelty produced curiosity and positive surprise for many participants.
What do you think about the addition of pumpkin to the beans?	Poor sensory fit reduces liking	Strong negative reactions	Not a good combination; Bittersweet; Least preferred	“Least favorite, not tasting good.” (FG2, P5); “Not a good combination.” (FG4, P2)	Taste, texture, and color concerns led many participants to dislike this combination.
What is your willingness to make these combinations for your family?	Positive sensory experience predicts adoption	Strong willingness to make sweet-potato-and-beans	Will cook; Most preferred; Appealing	“I will cook the sweet potato dish.” (FG6, P2); “Definitely will prepare it.” (FG6, P5)	Sweet-potato-and-beans was the combination most likely to be adopted at home.
	Social factors shape adoption	Some hesitation due to family preference	Reluctant to change; Not used to the combination	“My family is reluctant to change.” (FG3, P10)	Family habits and cultural norms affect willingness to introduce new dishes into the household.

FG = Focus Group; P = Participant number.

## Data Availability

The original contributions presented in this study are included in the article. Further inquiries can be directed to the corresponding author.

## Abbreviations

The following abbreviations are used in this manuscript:

ANOVA	Analysis of Variance
CFSP	Cream-Fleshed Sweet Potato
FGD	Focus Group Discussion
HACCP	Hazard Analysis and Critical Control Point
KCDM	King Cetshwayo District Municipality
KZN	KwaZulu-Natal
NUC	Neglected and Underutilized Crop
OA	Overall Acceptability
SPSS	Statistical Package for the Social Sciences
SSA	sub-Saharan Africa
UDM	uMkhanyakude District Municipality
